# Hyaluronan Graft Copolymers Bearing Fatty-Acid Residues as Self-Assembling Nanoparticles for Olanzapine Delivery

**DOI:** 10.3390/pharmaceutics11120675

**Published:** 2019-12-12

**Authors:** Marco Paolino, Mariano Licciardi, Cristina Savoca, Gaetano Giammona, Laura Modica De Mohac, Annalisa Reale, Germano Giuliani, Hartmut Komber, Alessandro Donati, Gemma Leone, Agnese Magnani, Maurizio Anzini, Andrea Cappelli

**Affiliations:** 1 Dipartimento di Biotecnologie, Chimica e Farmacia (Dipartimento di Eccellenza 2018-2022), Università degli Studi di Siena, Via A. Moro 2, 53100 Siena, Italy; reale5@student.unisi.it (A.R.); giuliani5@unisi.it (G.G.); alessandro.donati@unisi.it (A.D.); gemma.leone@unisi.it (G.L.); agnese.magnani@unisi.it (A.M.); maurizio.anzini@unisi.it (M.A.); andrea.cappelli@unisi.it (A.C.); 2 Dipartimento di Scienze e Tecnologie Biologiche, Chimiche e Farmaceutiche (STEBICEF), Università degli Studi di Palermo, Via Archirafi 32, 90123 Palermo, Italy; mariano.licciardi@unipa.it (M.L.); savocacristina@libero.it (C.S.); gaetano.giammona@unipa.it (G.G.); 3 Dipartimento Promozione della Salute, Materno-Infantile, di Medicina Interna e Specialistica di Eccellenza “G. D’Alessandro” (PROMISE), Università degli Studi di Palermo, 90100 Palermo, Italy; laura.modicademohac@unipa.it; 4 Leibniz Institute of Polymer Research, Hohe Strasse 6, 01069 Dresden, Germany; komber@ipfdd.de

**Keywords:** hyaluronic acid, ferulic acid, oleic acid, stearic acid, olanzapine, self-assembling nanocarriers, drug delivery systems

## Abstract

In order to evaluate the potential of a technology platform based on hyaluronan copolymers grafted with propargylated ferulate fluorophores (HA-FA-Pg) in the development of drug delivery systems, the propargyl groups of HA-FA-Pg derivatives were employed with oleic acid (OA) or stearic acid (SA) residues across a biocompatible hexa(ethylene glycol) (HEG) spacer. The designed materials (i.e., **HA-FA-HEG-OA** or **HA-FA-HEG-SA**) showed clear-cut aggregation features in an aqueous environment, as confirmed by dynamic light scattering (DLS) and transmission electron microscopy (TEM), generating nanoaggregate systems. In fact, **HA-FA-HEG-OA** and **HA-FA-HEG-SA** derivatives showed the property to create self-assembled cytocompatible nanostructured aggregates in water, thanks to the simultaneous presence of hydrophilic portions in the polymeric backbone, such as hyaluronic acid, and hydrophobic portions in the side chains. Furthermore, the designed materials interact with living cells showing a high degree of cytocompatibility. The potential ability of nanosystems to load pharmacologically active molecules was assessed by the physical entrapment of olanzapine into both polymeric systems. The drug loading evaluation demonstrated that the nanoparticles are able to incorporate a good quantity of olanzapine, as well as improve drug solubility, release profile, and cytocompatibility.

## 1. Introduction

Hyaluronic acid (HA, hyaluronan) is a glycosaminoglycan, which possesses important functions in the human body such as the formation of a pericellular coat having a crucial role in the early stages of cell adhesion [1]. In fact, HA interacts with the cluster of differentiation 44 (CD44) receptor [2,3] (a ubiquitous protein expressed in high density in tumor tissues [4]) showing an extracellular hyaluronan-binding domain.

**Figure 1:**
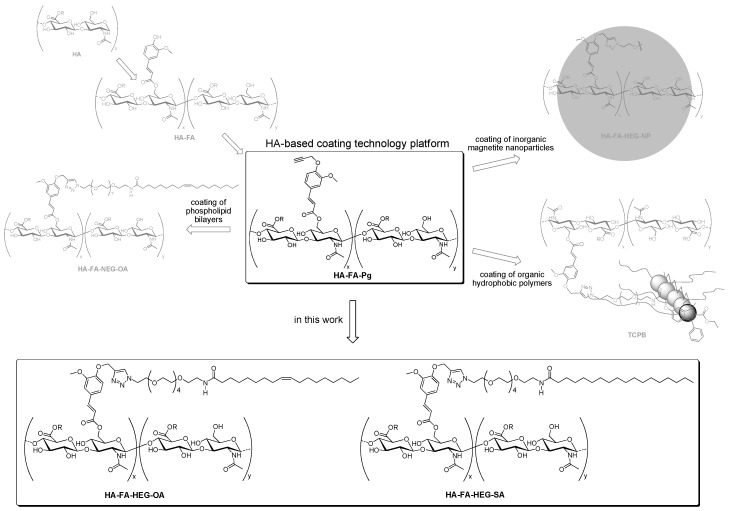
Design and development of the technology platform based on hyaluronan copolymers grafted with propargylated ferulate fluorophores (HA-FA-Pg).

In our previous studies, a synthetic procedure was developed to functionalize HA with ferulic acid (FA) residues in order to obtain graft copolymers (i.e., HA-FA derivatives, Figure 1) showing intriguing gelating and wound healing properties [5,6].

Subsequently, the grafting procedure was modified by using a ferulate (FA) derivative bearing a propargyl group, in order to introduce clickable groups to be employed in click-chemistry coupling reactions such as copper(I)-catalyzed azide–alkyne 1,3-dipolar cycloaddition (CuAAC) [7,8,9,10]. The procedure was optimized by using low-molecular-weight HA (molecular weight (M_W_) of about 8.7 kg∙mol^−1^), which provided hyaluronan copolymers grafted with propargylated ferulate fluorophores (HA-FA-Pg) showing grafting degree values in the range from 10% to 35% [11].

HA-FA-Pg derivatives were employed as the basis of a covalent coating technology with HA. In particular, an HA-FA-Pg derivative with 2–3 FA residues bearing a propargyl group was successfully used in the covalent coating of inorganic magnetite nanoparticles [12]. Moreover, an HA-FA-Pg derivative bearing 4–5 FA residues was used in the covalent coating of a π-stacked polybenzofulvene derivative [13,14,15,16,17,18,19] to prepare the intriguing organic material TCPB (Figure 1) [20].

In TCPB architecture, HA plays the role of the coating agent for both the polybenzofulvene cylindrical brush and the gel nanoparticles generated by TCPB aggregation in the water environment [20,21]. Interestingly, the gel nanoparticles were demonstrated to behave as intriguing carriers, capable of transporting the anticancer drug doxorubicin to cancer cells by exploiting the interaction of HA with the CD44 receptor overexpressed on the cell surface of tumor tissues [21]. The core of the nanogel systems generated by TCPB is constituted by the highly hydrophobic polybenzofulvene backbone and by the amphiphilic nona(ethylene glycol) (NEG) side chains that were assumed to form suitable compartments for drug loading, transport, and delivery.

However, in such architecture, the major potential drawback could be envisaged in the high hydrophobicity of the covalent backbone of the polybenzofulvene brush. Thus, in order to overcome this potential drawback, we envisioned the replacement of the covalent synthetic polybenzofulvene backbone of TCPB with oleic acid (OA), potentially capable of establishing networks in an aqueous environment by means of non-covalent interactions. These considerations led to the design of the tetra-component materials HA-FA-NEG-OA (Figure 1), which showed very intriguing features [11].

In particular, HA-FA-NEG-OA derivatives showed the tendency to generate nanostructured aggregates in water, which were cytocompatible and could be visualized by fluorescence microscopy techniques by virtue of the AIE (aggregation-induced emission) features of the ferulate luminogen [11]. Moreover, HA-FA-NEG-OA materials are capable of interacting with the phospholipid bilayer of monolamellar liposomes with an increase in the negative zeta potential without alterations in the liposome dimensions and the fluorogenic properties of the ferulate fluorophore [11]. On the basis of these promising results, HA-FA-NEG-OA materials could not only find applications in the coating of hydrophobic surfaces, but also in the formation of micellar systems potentially useful in the design of drug delivery systems [22,23,24].

Because of the multi-component architecture, the features of these materials could be modulated by the suitable variation of each component: (a) the polysaccharide chain, (b) the fluorophore, (c) the spacer, and (d) the fatty acid.

In the present paper, we describe the effects of the modification of two components of the previously described HA-FA-NEG-OA materials, namely, the spacer and the fatty acid. In particular, the nona(ethylene glycol) spacer was replaced with a more readily available hexa(ethylene glycol) (HEG) spacer, and the monounsaturated oleic acid (OA) residues were replaced with saturated stearic acid (SA) residues, thus producing **HA-FA-HEG-OA** and **HA-FA-HEG-SA** materials (Figure 1). The designed materials showed clear-cut aggregation features in an aqueous environment, highlighted by dynamic light scattering (DLS) and transmission electron microscopy (TEM) analysis, as well as the potential to act as cytocompatible nanocarriers for active molecules. In this study, olanzapine (OZ) was tested as a model drug [25,26].

## 2. Materials and Methods

The reagents and solvents were obtained from Sigma-Aldrich and were used as received. Olanzapine (OZ, M_W_ 312.43 g/mol) was purchased from Merck KGaA (Darmstadt, Germany). Ethanol, Dulbecco’s phosphate-buffered saline (DPBS), anhydrous dimethyl sulfoxide (DMSO), sodium chloride, and sodium hydroxide were obtained from Merck KGaA (Darmstadt, Germany). The water used was produced with Milli-Q ^®^ (Millipore Corporation, Billerica, MA, USA). Merck silica gel 60 (230–400 mesh) was employed for column chromatography, and Merck thin layer chromatography (TLC) aluminum sheets (silica gel 60 F_254_) were employed for TLC. NMR spectra were obtained with a Bruker DRX-400 AVANCE or a Bruker Avance III 500 spectrometer in the indicated solvents. The chemical shifts are referenced to the solvent signal (CDCl_3_: δ(^1^H) = 7.26 ppm, δ(^13^C) = 77.0 ppm) or to the signal of a trace acetone for solutions in D_2_O (δ(^1^H) = 2.22 ppm, δ(^13^C)_CH3_ = 30.9 ppm). Chemical shifts (δ) are reported in ppm, and the H–H coupling constants (*J*) are reported in Hz. An Agilent 1100 LC/MSD running with an electrospray source was used in mass spectrometry measurements. The intermediate α-azido-ω-amino hexa(ethylene glycol) **3** was synthesized as previously reported in Reference [27].

### 2.1. Synthesis and Characterization

#### *N*-(17-Azido-3,6,9,12,15-pentaoxaheptadecyl)oleamide (4a)

A mixture of oleic acid (**1a**, 0.34 g, 1.20 mmol) and1,1′-carbonyldiimidazole (CDI) (0.20 g, 1.23 mmol) in dry tetrahydrofuran (THF) (5.0 mL) was refluxed for 2 h, and then a solution *O*-(2-aminoethyl)-*O*′-(2-azidoethyl)tetraethylene glycol (**3**, 0.40 g, 1.31 mmol) in dry THF (5.0 mL) was added. The resulting mixture was stirred overnight at room temperature and then concentrated under reduced pressure. The resulting oily residue was purified by flash chromatography with ethyl acetate to obtain 4a as a pale-yellow oil (0.65 g, yield 95%). ^1^H-NMR (400 MHz, CDCl_3_): 0.87 (t, *J* = 6.6, 3H), 1.24–1.31 (20H), 1.62 (m, 2H), 1.92–2.05 (4H), 2.16 (t, *J* = 7.6, 2H), 3.37 (t, *J* = 5.1, 2H), 3.44 (q, *J* = 5.1, 2H), 3.54 (t, *J* = 5.1, 2H), 3.59–3.74 (18H), 5.24–5.43 (2H), 6.05 (br s, 1H). MS (ESI): *m*/*z* 593.2 (M + Na^+^).

#### *N*-(17-Azido-3,6,9,12,15-pentaoxaheptadecyl)stearamide (4b)

A mixture of stearic acid (1b, 0.18 g, 0.63 mmol) and CDI (0.10 g, 0.62 mmol) in dry THF (10 mL) was refluxed for 2 h, and then a solution of *O*-(2-aminoethyl)-*O*′-(2-azidoethyl)tetraethylene glycol (3, 0.20 g, 0.65 mmol) in dry THF (5.0 mL) was added. The resulting mixture was stirred overnight at room temperature and then concentrated under reduced pressure. The resulting oily residue was purified by flash chromatography with petroleum ether/ethyl acetate (1:1) to obtain 4b as a white solid (0.33 g, yield 93%, melting point (mp) 48–50 °C). ^1^H-NMR (400 MHz, CDCl_3_): 0.87 (t, *J* = 6.7, 3H), 1.17–1.38 (28H), 1.60 (m, 2H), 2.15 (t, *J* = 7.6, 2H), 3.37 (t, *J* = 5.1, 2H), 3.43 (q, *J* = 5.3, 2H), 3.54 (t, *J* = 5.0, 2H), 3.58–3.72 (18H), 6.02 (br s, 1H). MS (ESI): *m*/*z* 595.2 (M + Na^+^).

#### Preparation of **HA-FA-HEG-OA**

A round-bottom 10-mL flask was charged, under an inert atmosphere, with *tert*-butanol (2.0 mL), H_2_O (2.0 mL), and a solution of copper sulfate pentahydrate (12.5 mg, 0.050 mmol in 0.50 mL of water). Subsequently, a 1 M water solution of sodium ascorbate (0.50 mL) was added. A portion of the resulting catalyst solution (1.0 mL) was promptly used in the subsequent reaction.

To a mixture of HA-FA-Pg graft copolymer bearing a mean distribution of 4–5 propargyl derivatives for each macromolecule (200 mg, synthetic procedure reported in Reference [11]) in 5.0 mL of water containing 4a (60 mg, 0.105 mmol), 1.0 mL of the catalyst solution was added, and the resulting mixture was stirred at room temperature for 1 h and then treated with QUADRASIL MP (200 mg). After filtration, the resulting mixture was concentrated under reduced pressure. The solid residue was purified by washing with acetone to obtain pure **HA-FA-HEG-OA** as a light-brown solid (218 mg). ^1^H-NMR (500 MHz, D_2_O): the spectrum is depicted in Figure 3c; ^13^C-NMR (125 MHz, D_2_O), selected signals: 14.6 (18″), 23.2 (17″, Et), 26.4 (3″), 27.8 (8″, 11″), 29–32 (4″–7″, 12″–15″), 32.5 (16″), 36.6 (2″), 39.6 (H), 50.8 (E), 69–72 (F,G,X,Y), 130.0 and 130.4 (9″,10″), 175.6 (1″). For the spectrum and the atom numbering, compare Figure 4d.

#### Preparation of **HA-FA-HEG-SA**

Under an inert atmosphere, a round-bottom 10-mL flask was charged with *tert*-butanol (2.0 mL), H_2_O (2.0 mL), and a solution of copper sulfate pentahydrate (12.5 mg, 0.050 mmol in 0.50 mL of water). Subsequently, a 1 M water solution of sodium ascorbate (0.50 mL) was added. A portion of the resulting catalyst solution (1.0 mL) was promptly used in the subsequent reaction.

To a mixture of HA-FA-Pg graft copolymer bearing a mean distribution of 4–5 propargyl derivatives for each macromolecule (200 mg, synthetic procedure reported in Reference [11]) in water (5.0 mL) containing 4b (62 mg, 0.108 mmol), 1.0 mL of the catalyst solution was added, and the resulting mixture was stirred at room temperature for 1 h and then treated with QUADRASIL MP (200 mg). After filtration, the resulting mixture was concentrated under reduced pressure. The solid residue was purified by washing with acetone to obtain **HA-FA-HEG-SA** as a light-brown solid (230 mg). ^1^H-NMR (500 MHz, D_2_O): the spectrum is depicted in Figure 3d; ^13^C-NMR (125 MHz, D_2_O), selected signals: 14.6 (18″), 23.2 (17″,Et), 26.4 (3″), 29–32 (4″–15″), 32.6 (16″), 36.6 (2″), 39.7 (H), 69–72 (F,G,X,Y), 175.6 (1″). For the spectrum and the atom numbering, compare Figure 4c.

#### (*E*)-Methyl 3-[3-methoxy-4-[[1-[(*Z*)-19-oxo-3,6,9,12,15-pentaoxa-18-azahexatriacont-27-en-1-yl]-1*H*-1,2,3-triazol-4-yl]methoxy]phenyl]acrylate (FE-HEG-OA)

Under an inert atmosphere, a round-bottom 10-mL flask was charged with *tert*-butanol (2.0 mL), H_2_O (2.0 mL), and a solution of copper sulfate pentahydrate (12.5 mg, 0.050 mmol in 0.50 mL of water). Then, a 1 M water solution of sodium ascorbate (0.50 mL) was added. A portion of the resulting catalyst solution (1.0 mL) was promptly used in the subsequent reaction. A mixture of 4a (250 mg, 0.44 mmol) and 5 [20] (110 mg, 0.45 mmol) in 4.0 mL of *tert*-butanol and water (2.0 mL) was treated with the catalyst solution (2.7 mL), and the reaction mixture was stirred overnight at room temperature and then concentrated under reduced pressure. Purification of the residue by flash chromatography with ethyl acetate/methanol (9:1) as the eluent afforded **FE-HEG-OA** as a pale-yellow oil (337 mg, yield 92%). ^1^H-NMR (500 MHz, CDCl_3_): 0.88 (t, *J* = 6.9, 3H; 18″), 1.2–1.35 (20H; 4″–7″,12″–17″), 1.62 (m, 2H; 3″), 2.00 (4H; 8″,11″), 2.16 (t, *J* = 7.6, 2H; 2″), 3.43 (q, *J* = 5.3, 2H; H), 3.54 (t, *J* = 5.1, 2H; G), 3.55–3.65 (16H; X,Y), 3.80 (s, 3H; A″), 3.87 (t, *J* = 5.0, 2H; F), 3.89 (s, 3H; A), 4.53 (t, *J* = 5.0, 2H; E), 5.3–5.35 (4H; 9″,10″,B), 6.05 (br, 1H; NH), 6.31 (d, *J* = 16.0, 1H; 2), 7.05–7.10 (3H; 2′,5′,6′), 7.62 (d, *J* = 16.0, 1H; 3), 7.83 (s, 1H; D). ^13^C NMR (125 MHz, CDCl_3_): 14.1 (18″), 22.7 (17″), 25.7 (3″), 27.2 (8″,11″), 29.2–29.7 (4″–7″,12″–15″), 31.9 (16″), 36.7 (2″), 39.1 (H), 50.4 (E), 51.6 (A’), 55.9 (A), 63.0 (B), 69.4 (F), 70.0–70.5 (F,G,X,Y), 110.3 (2′), 113.7 (5′), 115.9 (2), 122.4 (6′), 124.3 (D), 128.1 (1′), 129.7 and 130.0 (9″,10″), 143.5 (C), 144.6 (3), 149.7, and 149.8 (3′,4′), 167.6 (1), 173.2 (1″). For spectra and the atom numbering, compare Figures 3e and 4e. MS (ESI): *m*/*z* 839.5 (M + Na^+^).

#### (*E*)-3-[3-Methoxy-4-[[1-[(*Z*)-19-oxo-3,6,9,12,15-pentaoxa-18-azahexatriacont-27-en-1-yl]-1*H*-1,2,3-triazol-4-yl]methoxy]phenyl]acrylic acid (**FA-HEG-OA**)

**Figure 2:**
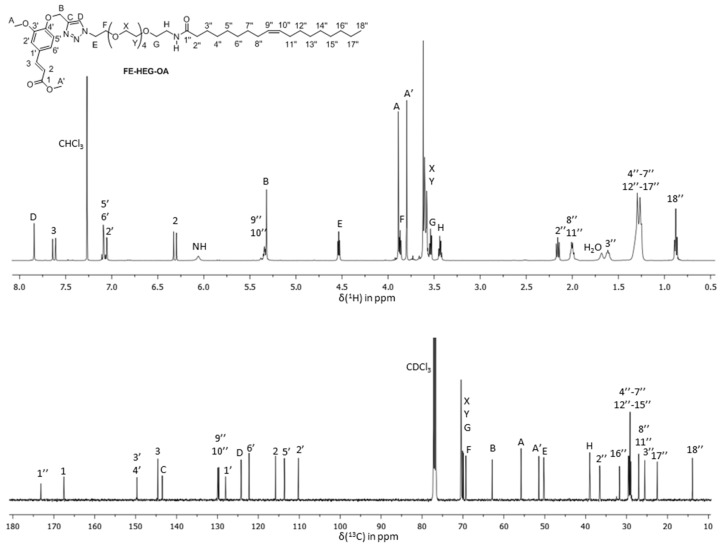
^1^H- and ^13^C-NMR spectra of model compound **FA-HEG-OA** in CDCl_3_.

A mixture of **FE-HEG-OA** (0.33 g, 0.40 mmol) in ethanol (7.5 mL) containing a 2 N water solution of NaOH (4.7 mL) was refluxed for 2 h. The reaction mixture was then cooled at 0 °C, acidified with 3 N HCl, and concentrated under reduced pressure. Purification of the residue by flash chromatography with dichloromethane/methanol (9:1) gave acid **FA-HEG-OA** as a white solid (0.28 g, yield 87%, mp 67–69 °C). ^1^H-NMR (500 MHz, CDCl_3_): 0.88 (t, *J* = 6.9, 3H; 18″), 1.2–1.35 (20H; 4″–7″,12″–17″), 1.62 (m, 2H; 3″), 2.00 (4H; 8″,11″), 2.17 (t, *J* = 7.6, 2H; 2″), 3.44 (q, *J* = 5.3, 2H; H), 3.5–3.65 (18H; G,X,Y), 3.86 (t, *J* = 5.0, 2H; F), 3.90 (s, 3H; A), 4.53 (t, *J* = 5.0, 2H; E), 5.3–5.4 (4H; 9″,10″,B), 6.12 (br, 1H; NH), 6.31 (d, *J* = 16.0, 1H; 2), 7.05–7.10 (3H; 2′,5′,6′), 7.68 (d, *J* = 16.0, 1H; 3), 7.81 (s, 1H; D), 10.60 (br, 1H; COOH). ^13^C NMR (125 MHz, CDCl_3_): 14.1 (18″), 22.6 (17″), 25.7 (3″), 27.2 (8″,11″), 29.1–29.7 (4″–7″,12″–15″), 31.9 (16″), 36.7 (2″), 39.1 (H), 50.4 (E), 55.9 (A), 62.9 (B), 69.4 (F), 70.0–70.5 (F,G,X,Y), 110.4 (2′), 113.7 (5′), 115.6 (2), 122.7 (6′), 124.3 (D), 127.8 (1′), 129.7 and 130.0 (9″,10″), 143.5 (C), 146.2 (3), 149.7, and 150.0 (3′,4′), 170.9 (1), 173.4 (1″). For spectra and the atom numbering, compare Figure 2. MS (ESI): *m*/*z* 825.5 (M + Na^+^).

### 2.2. Particle Size Analysis and Zeta Potential

The size analysis, zeta potential, and polydispersity index of the nanoparticles (NPs) were determined using a Malvern Zetasizer Nano ZS instrument, fitted with a 532-nm laser at a fixed scattering angle of 90°. The samples were analyzed before and after drug loading. Each sample was suitably diluted and filtered before the analysis and placed in a disposable sizing micro cuvette.

### 2.3. Critical Aggregation Concentration

Critical aggregation concentration (CAC) evaluation was carried out in water and in DPBS by means of the pyrene fluorescence assay. At the beginning, a solution of pyrene in acetone was prepared (6.0 × 10^−5^ M); then, aliquots of 20 μL of this solution were placed in vials and left to evaporate for 30 min in orbital shaker at 37 °C in order to remove the acetone. Afterward, 2 mL of the polymer aqueous dispersions were added to the pyrene solid residue. Polymer concentrations were included in a range from 10 to 0.0005 mg/mL. In this way, the final concentration of pyrene in each sample was, thus, equal to 6.0 × 10^−7^ M. The mixtures were kept at 37 °C for 1 h under continuous agitation, inside an orbital shaker, in order to balance the pyrene with the micelles. The pyrene excitation and emission spectra were recorded at 25 °C at an excitation wavelength of 333 nm and an emission wavelength of 373 nm.

### 2.4. Transmission Electron Microscopy (TEM) Analysis

In order to characterize morphology and size of NPs, a TEM analysis was carried out. Samples were visualized using an electronic microscope JEOL JEM-2100 at 100 KV, and Digital Micrograph Gatan (Gatan, Inc., Pleasanton, CA, USA) was used as the software. Samples (1.0 mg) were solubilized in water (1.0 mL). A drop of each sample was placed in a carbon-coated grid and dried overnight before analysis.

### 2.5. Rheological Analysis

Firstly, 2% *w*/*v* **HA-FA-HEG-SA** and HA (M_W_ 8700 g·mol^−1^) solutions were prepared in bi-distilled water. Flow curves were recorded using an HR-2 Hybrid-Rheometer (TA-Instruments, Leatherhead, United Kingdom) with a cone-plate measuring device 40 mm in diameter, employing a gap size of 0.1 mm. The temperature was maintained at 25 °C. The dynamic viscosity η under a controlled shear rate ranging from 1 to 100 s^−1^ was measured [28].

### 2.6. Preparation of Self-Assembling Nanoparticles

Olanzapine (OZ, M_W_ 312.43 g/mol) was purchased from Merck KGaA (Darmstadt, Germany). Ethanol, Dulbecco’s phosphate-buffered saline (DPBS), anhydrous dimethyl sulfoxide (DMSO), sodium chloride, and sodium hydroxide were obtained from Merck KGaA (Darmstadt, Germany). The water used was produced with Milli-Q ^®^ (Millipore Corporation, Billerica, MA, USA).

Aliquots of **HA-FA-HEG-OA** (30 mg) and **HA-FA-HEG-SA** (30 mg) were dissolved in 3.0 mL of Milli-Q water and left, under vigorous stirring, at room temperature overnight. The day after, OZ (15 mg) was added to polymer dispersions, to get a drug concentration of 5 mg/mL, and the mixture was left under vigorous stirring, at room temperature overnight. After this time, the dispersions were centrifuged at 8000 rpm and 25 °C for 5 min to remove the drug, which was not incorporated in the self-assembling nanoparticles (NPs). Supernatant was recovered and then dried by freeze-drying, thereby obtaining a spongy solid.

### 2.7. Drug Content and Encapsulation Efficiency

The drug content in the NP was determined by dissolving 2.0 mg of the obtained dried products in 2.0 mL of anhydrous DMSO. Absorbance of the solutions was then measured spectrophotometrically at 276 nm after appropriate dilution 1:10 in anhydrous DMSO, and the drug content in the NP was determined. Drug loading (DL) in the NP was calculated by using the calibration curve, built in a range between 0.05 and 0.001 mg/mL, as follows:

DL (%) = A/B × 100,

where A is the drug content in the NP, and B is the weight of NP. The results were confirmed taking into consideration the polymer’s interference in the analysis.

### 2.8. Drug Release Studies

The release of OZ from **HA-FA-HEG-OA** and **HA-FA-HEG-SA** NP was evaluated using the dialysis bag diffusion technique. The release studies were performed in Dulbecco’s phosphate-buffered saline (DPBS) (pH 7.4) mimicking plasma compartment pH conditions, with 3% (*v*/*v*) Tween-80 to create a perfect sink condition, since OZ has limited solubility in the buffer. The aqueous NP dispersion equivalent to 0.7 mg of OZ was placed into a dialysis bag (cut-off 3.5–5 kDa; Spectra por, Flot-A-Lyzer G2 Dialysis Device). The dialysis bag was immersed in the acceptor compartment containing 50 mL of DPBS pH 7.4 plus 3% *v*/*v* Tween-80, and incubated under continuous stirring (100 rpm) and maintained at 37 °C in an orbital shaker incubator for 30 h. The acceptor compartment was covered to prevent the evaporation. At regular time intervals, specific volumes of the external medium were withdrawn, and the same volume was replaced by fresh buffer. The withdrawn samples were centrifuged and then freeze-dried. The solid residues were dissolved in DMSO and analyzed spectrophotometrically at 276 nm. The amount of released OZ was determined by using a calibration curve, built in a range between 0.05 and 0.001 mg/mL. The released OZ was determined as a function of incubation time, and it is reported as the weight percentage of the total amount of drug loaded into the polymer aggregates. The dilution procedure was considered in the correction of the data.

### 2.9. Cytotoxicity Assay

Cell viability was assessed on human bronchial epithelial cells (16HBE) by the tetrazolium salt (MTS) assay. Cells were grown in Dulbecco’s modified Eagle’s medium (DMEM) with 10% FBS (fetal bovine serum) and 1% penicillin/streptomycin (100 U∙mL^−1^ penicillin and 100 µg∙mL^−1^ streptomycin), at 37 °C in a 5% CO_2_ humidified atmosphere after seeding in a 96-well plate at a density of 2 × 10^4^ cells per well. Different amounts of nanosystems (i.e., empty and OZ-loaded **HA-FA-HEG-OA** and **HA-FA-HEG-SA**) and free OZ as control were weighed in order to obtain drug concentrations corresponding to 350 µM, 200 µM, 100 µM, and 50 µM. The empty and OZ-loaded systems were dispersed in DMEM, while OZ was dissolved in DMSO because of the low drug solubility in water. Aliquots of the sample dispersions (200 µL) were added to the cells and incubated for 24 h. After incubation, in each well, the medium was replaced with 100 µL of fresh DMEM and 20 µL of MTS (3-(4,5-dimethylthiazol-2-yl)-5-(3-carboxymethoxyphenyl)-2-(4-sulfophenyl)-2H-tetrazolium) reagent solution. Plates were incubated at 37 °C in a 5% CO_2_ humidified atmosphere for 1 h. Absorbance was measured by a multi-well plate reader (Eppendorf AF 2200) at 490 nm. All the experiments were performed in triplicate, and average values were calculated. The equation, (Abs490 treated cells/Abs490 control cells) × 100, was used to calculate the relative cell viability (percentage) as an average of three values. A positive control was obtained with untreated cells.

### 2.10. Statistical Analysis

A one-way analysis of variance (ANOVA) was applied to compare different samples. Data were considered statistically significant with a value of *p* < 0.05, and differences between groups were compared using the Bonferroni *t*-test. Each test was developed in triplicate.

## 3. Results and Discussion

### 3.1. Synthesis of **HA-FA-HEG-OA** and **HA-FA-HEG-SA** Materials

**Scheme 1:**
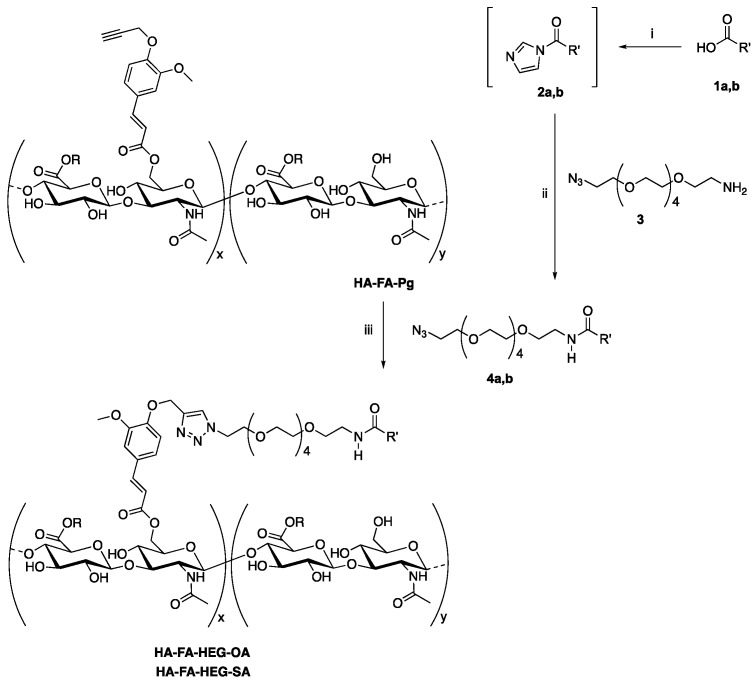
Synthesis of **HA-FA-HEG-OA** and **HA-FA-HEG-SA** materials. Reagents: (**i**) CDI, THF; (**ii**) TEA, THF; (**iii**) CuSO_4_, sodium ascorbate, *tert*-BuOH, H_2_O. Substituents: R = H or C_2_H_5_; R’ = C_17_H_33_ (1a, 2a, 4a, **HA-FA-HEG-OA**) or C_17_H_35_ (1b, 2b, 4b, **HA-FA-HEG-SA**).

**HA-FA-HEG-OA** and **HA-FA-HEG-SA** derivatives were prepared from the previously published HA-FA-Pg derivative, bearing a mean distribution of 4–5 propargyl derivatives for each macromolecule [11] by CuAAC coupling [29] with the suitable α-azido-ω-fatty amide hexa(ethylene glycol) (azido-HEG-OA, 4a or azido-HEG-SA, 4b) as shown in Scheme 1.

In particular, the appropriate fatty acid (OA, 1a or SA, 1b) was activated with 1,1′-carbonyldiimidazole (CDI) in refluxing THF to obtain the corresponding imidazolide (2a,b), which was promptly reacted with α-azido-ω-amino hexa(ethylene glycol) (3) to obtain the required azido-HEG-OA (4a) or azido-HEG-SA (4b). The CuAAC coupling was performed under very mild conditions with the copper(I) catalyst being generated in situ with CuSO_4_/sodium ascorbate. Moreover, the coupling reaction was carried out by using HA-FA-Pg samples obtained from low-molecular-weight HA (M_W_ about 8.7 kg∙mol^−1^) and showing a degree of grafting around 20%, corresponding to four propargyl groups per HA macromolecule in order to prepare materials bearing about four fatty-acid side chains pending from the HA backbone [11].

**Scheme 2:**
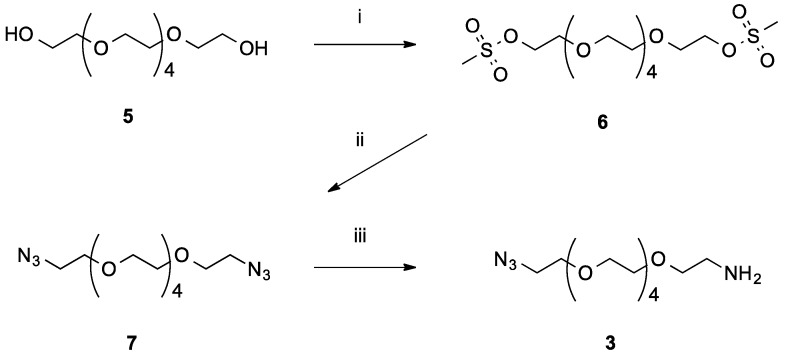
Synthesis of hexa(ethylene glycol) derivative 3. Reagents: (**i**) MesCl, TEA, CH_2_Cl_2_; (**ii**) NaN_3_, Bu_4_N^+^Br^−^, DMF; (**iii**) PPh_3_, toluene, HCl 3 N.

The intermediate α-azido-ω-amino hexa(ethylene glycol) [27] (3) was synthesized as described in Scheme 2 and previously reported in Reference [27].

**Scheme 3:**
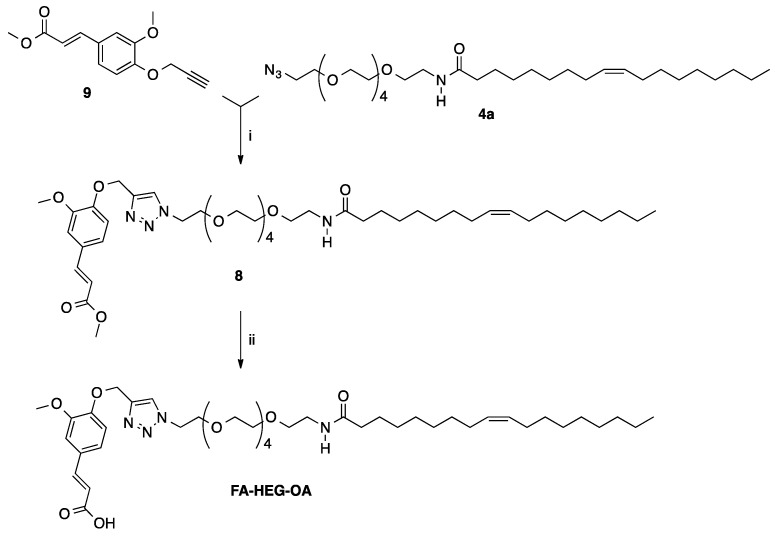
Synthesis of model compounds 8 and **FA-HEG-OA**. Reagents: (**i**) CuSO_4_, sodium ascorbate, *tert*-BuOH, H_2_O; (**ii**) NaOH, H_2_O, C_2_H_5_OH.

Finally, compound **FA-HEG-OA** and its methyl ester precursor 8 were synthesized from ferulate derivative 9 [11] and compound 4a as described in Scheme 3 in order to be used as model compounds.

### 3.2. Structure of **HA-FA-HEG-OA** and **HA-FA-HEG-SA** Materials

**Figure 3:**
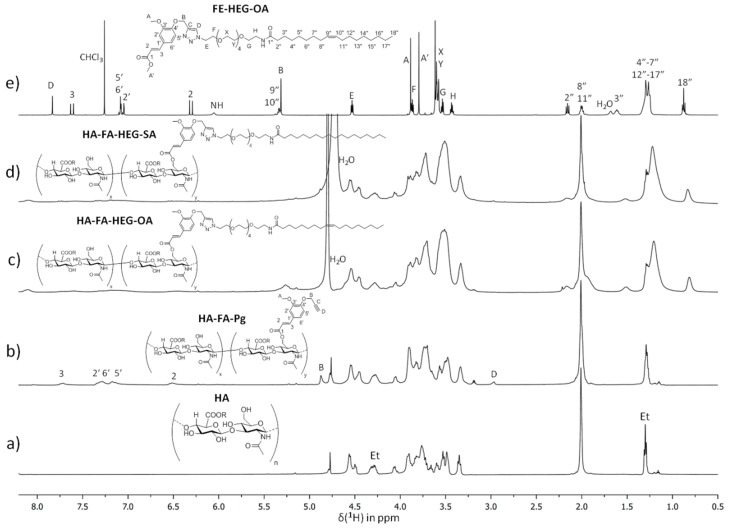
Comparison of ^1^H-NMR spectra obtained from **HA-FA-HEG-OA** (**c**) and **HA-FA-HEG-SA** (**d**) derivatives measured in D_2_O and **FE-HEG-OA** (**e**) measured in CDCl_3_ with those obtained from the starting HA-FA-Pg (**b**) and HA (**a**) samples (solvent: D_2_O; (**a**) and (**b**) were recorded with water suppression). In the spectrum of HA, “Et” labels indicate the signals of ethyl groups of the monomeric units with R = C_2_H_5_.

**Figure 4:**
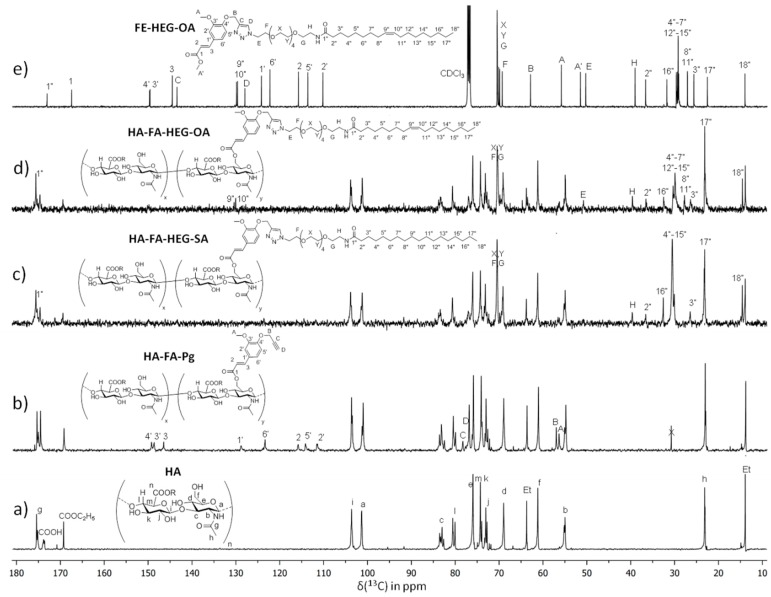
Comparison of ^13^C-NMR spectra of **HA-FA-HEG-SA** (**c**) and **HA-FA-HEG-OA** (**d**) derivatives measured in D_2_O and **FE-HEG-OA** (**e**) measured in CDCl_3_ with those obtained from starting HA-FA-Pg (**b**) and HA (**a**) samples in D_2_O. In the spectrum of HA, “Et” labels indicate the signals of ethyl groups of the monomeric units with R = C_2_H_5_.

The structure of **HA-FA-HEG-OA** and **HA-FA-HEG-SA** derivatives was studied by ^1^H- and ^13^C-NMR spectroscopy in D_2_O as the solvent (Figure 3 and Figure 4).

In a first step, the assignment of the ^1^H- and ^13^C-NMR spectra of HA-FA-Pg was re-investigated in order to evaluate previous assignments [11,20]. In particular, we focused on the signals of the propargyl group, which are considered diagnostic signals for evaluating the functionalization of the HA-FA-Pg copolymer. Combing one-dimensional (1D) and two-dimensional (2D) NMR methods, the signals of propargyl protons B and D, as well as of carbons B, C, and D, can be unequivocally assigned (Figure 3b and Figure 4b)

The intensity of the proton signals allows monitoring their conversion after the CuAAC click reaction. Thus, in order to ascertain the effectiveness of the CuAAC coupling reaction producing **HA-FA-HEG-OA** and **HA-FA-HEG-SA** derivatives, the ^1^H- and ^13^C-NMR spectra of these materials were compared with the corresponding spectra of HA and HA-FA-Pg (Figure 3 and Figure 4).

The conversion of the alkyne moiety of HA-FA-Pg into the triazole one of **HA-FA-HEG-OA** and **HA-FA-HEG-SA**, indicative of a successful CuAAC coupling reaction, was proven by the disappearance of the signal of acetylene proton D at 2.98 ppm observed in the ^1^H-NMR spectrum of HA-FA-Pg (Figure 3b) and the appearance of a new peak at around 8 ppm in the spectra of **HA-FA-HEG-OA** and **HA-FA-HEG-SA** (Figure 3c,d). Analogously, in the ^13^C-NMR spectra (Figure 4), the alkyne signal C at 78.5 ppm disappeared. This observation was first evidence of the covalent insertion of components HEG-OA or HEG-SA in the new materials. Moreover, a significant line broadening was observed for the signals assigned to the aromatic and acrylic protons of the FA moiety.

Owing to their complex structures, the ^1^H-NMR spectra of **HA-FA-HEG-OA** and **HA-FA-HEG-SA** (Figure 3) were composed by a hard-to-assign set of broad and partially overlapped signals of the different substructures, representing the hyaluronan backbone, FA triazole moiety, and PEG fatty acid functional group. However, the ^13^C-NMR spectra allowed a closer view of the chemical structures. In this context, two model substances, **FE-HEG-OA** (Figure 3e and Figure 4e) and FE-HEG-SA (Figure 2), were synthesized and characterized in detail by ^1^H- and ^13^C-NMR spectroscopy. As obvious from the comparison of ^13^C-NMR spectra in Figure 4, in addition to the dominating signals of the HA backbone, both the HEG signals in the 72–69 ppm region and the alky group signals (40 – 25 ppm, CH_3_ group at 14.6 ppm) appeared. For the OA derivative, the olefinic carbons resulted in two signals at 130.4 and 130.0 ppm.

Unlike the parent HA-FA-Pg, the signals of the aromatic carbon atoms of the ferulate–triazole structure were almost not detectable. In an attempt to explain this result, we assumed that the FA moiety suffered from reduced mobility, maybe due to poor solvation in D_2_O producing a pronounced line broadening as already observed in the ^1^H-NMR spectra in Figure 3. Here, one has to recall the disappearance of propargyl signals and the persistence of the signal assigned to the ferulate methoxy (at about 55.6 ppm). Consequently, by comparison of the NMR spectra of the new copolymer materials with those of the starting materials and synthesized model molecules, we successfully assigned all detectable signals of the complex spectra obtained for **HA-FA-HEG-OA** and **HA-FA-HEG-SA**.

### 3.3. Self-Assembling Properties

**HA-FA-HEG-OA** and **HA-FA-HEG-SA** were designed to be biocompatible and partially hydrophilic polymers showing clear-cut aggregation features in an aqueous environment. Thanks to their partially amphiphilic features, they are potentially capable of forming self-assembled nanoparticles in water dispersions and loading lipophilic drugs such as olanzapine, the solubility of which is rather negligible in physiological fluids. Moreover, the hydrophilic portion of the copolymer, which we assumed to be exposed on the surface of the nanosystems, can be used to provide the nanoparticles with stealth properties for longer circulation times.

In particular, **HA-FA-HEG-OA** and **HA-FA-HEG-SA** derivatives showed a tendency to create cytocompatible nanostructured aggregates in water, owing to the simultaneous presence of hydrophilic portions in the polymeric chain (i.e., hyaluronic acid, an essential component of connective tissues in humans that plays an essential role in the early stages of cell adhesion) and oleic or stearic hydrophobic portions. The resulting features of the polymeric nanocarriers, such as surface charge, dimensions, balance between hydrophilic and hydrophobic parts, and biocompatibility, confirmed the possibility of using these materials as drug delivery systems.

**Figure 5:**
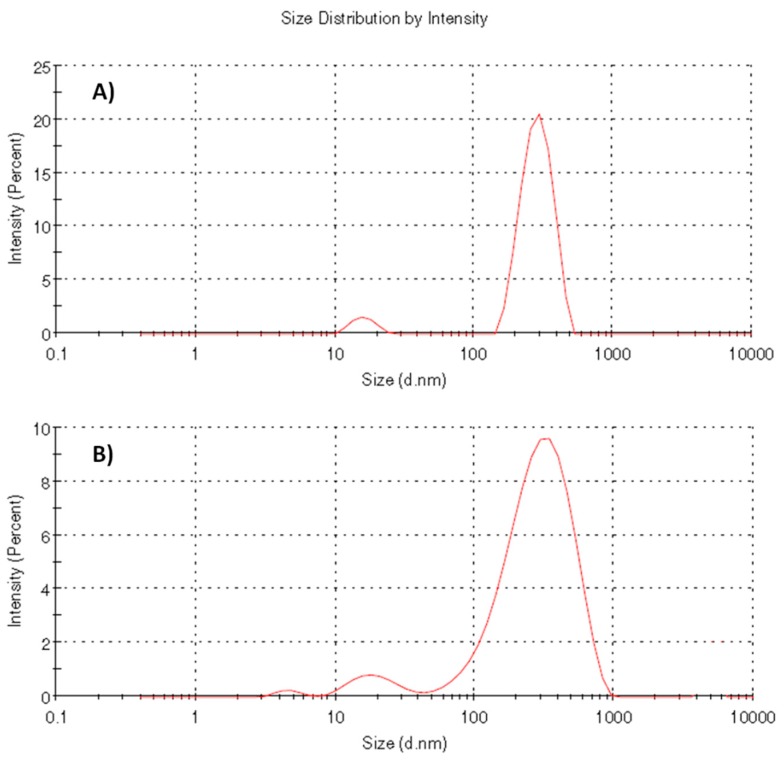
DLS size distribution histograms of **HA-FA-HEG-OA** (**A**) and **HA-FA-HEG-SA** (**B**) in Milli-Q water.

Polymers were simply dispersed in Milli-Q water in a concentration of 0.5 mg/mL and left to shake alternating with sonication cycles in order to unpack the macromolecules and favor dissolution. The systems were left to agitate overnight and then analyzed by DLS after filtration in order to evaluate both the presence of dispersed aggregates and the zeta potential of the particulate matter. The results shown in Table 1 confirmed the ability of **HA-FA-HEG-OA** and **HA-FA-HEG-SA** derivatives to self-assemble into NPs upon dispersion in water. However, DLS curves (Figure 5) revealed the presence of two heterogeneous populations: one showing a small mean diameter (below 20 nm) that was assumed to be constituted by small aggregates of a few macromolecules, and a second population with greater dimensions constituted by a large number of macromolecules.

Moreover, the negative surface charge (in the range −35 to −47 mV) shown by both systems represents a stabilizing factor owing to repulsion between NPs, preventing their aggregation.

In order to evaluate the potential critical aggregation concentration (CAC) of polymers in aqueous media, pyrene was used as the fluorescent hydrophobic probe. The high hydrophobicity of the probe should allow us to predict the ability of the system to internalize active molecules. This method is based on the difference in pyrene fluorescence when the probe is situated in a water solution or in the hydrophobic nucleus of micelles [30]. In particular, both the I1/I3 ratio obtained from the pyrene emission spectra and the I338/I332 ratio obtained from the pyrene excitation spectra, measured at 25 °C, were plotted versus the logarithm of the copolymer concentration. The obtained curves were used for the evaluation of the critical aggregation concentration (CAC) identifiable with the intersection of the tangent to the curve at the inflection with the horizontal tangent through the points at lower concentrations [31].

**Figure 6:**
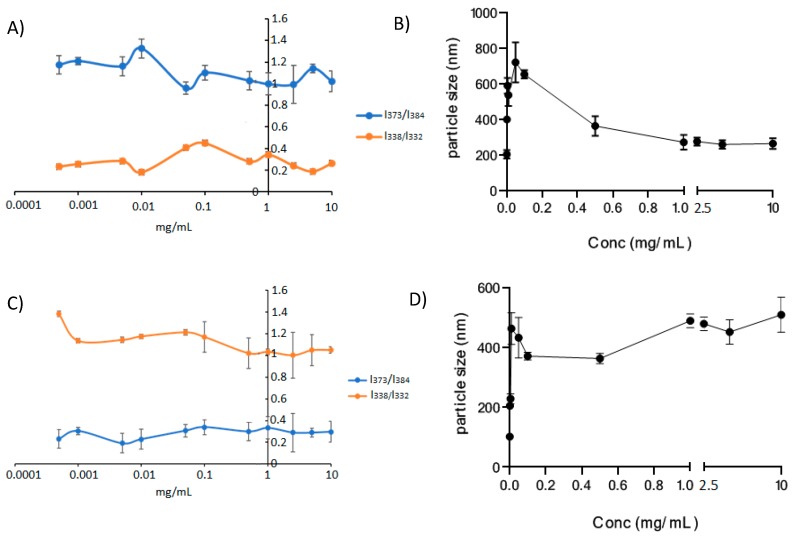
I1_(373)_/I3_(384)_ intensity ratios obtained from pyrene emission spectra (blue curve) and I338/I332 intensity ratios obtained from pyrene excitation spectra (orange curve) for **HA-FA-HEG-OA** derivative water dispersions (**A**) or **HA-FA-HEG-SA** derivative water dispersions (**C**) at 25 °C, in the concentration range of 0.0001–10 mg/mL; size distribution values of **HA-FA-HEG-OA** (**B**) or **HA-FA-HEG-SA** (**D**) in the same conditions.

As can be seen in the graph shown in Figure 6A, for the **HA-FA-HEG-OA** derivative, there was not a sufficiently significant change in the emission and excitation spectra to justify a concentration-dependent aggregation phenomenon. However, at the concentration of 0.5 mg/mL, DLS analysis (Figure 6B) showed the presence of colloidal particles with a size of 378 nm, which stabilized to the size of 270 nm at increasing concentrations between 1 and 10 mg/mL.

Also, in the case of the **HA-FA-HEG-SA** derivative (Figure 6C), we were unable to obtain a characteristic CAC value, but the results of DLS analysis in the concentration range between 0.5 and 10 mg/mL suggested the formation of colloidal particles of increasing size from 363 to 569 nm.

The results suggested that the materials do not form so-called micelles, but rather stable self-assembled nanoparticles.

**Figure 7:**
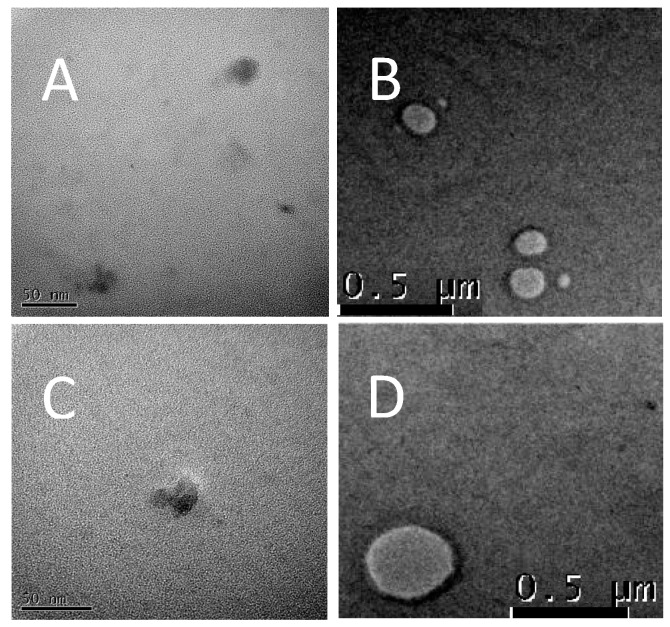
Representative transmission electron micrograph of **HA-FA-HEG-OA** (**A**,**B**) and **HA-FA-HEG-SA** (**C**,**D**) nanoparticles.

TEM observations, reported in Figure 7, showed that **HA-FA-HEG-OA** (Figure 7A,B) and **HA-FA-HEG-SA** (Figure 7C,D) nano-aggregates appeared as spherical objects, showing dimensions slightly smaller than those obtained by DLS measurements, due to the shrinkage of nano-aggregates upon dehydration. In line with the DLS results, TEM analysis showed the contemporary presence of small objects (diameter around 20 nm) and larger nano-aggregates.

**Figure 8:**
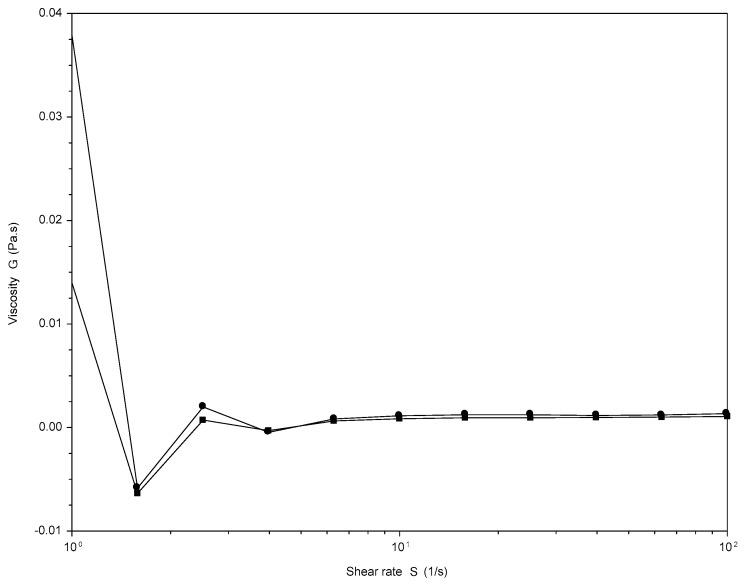
Viscosity curve of 2% *w*/*v* aqueous solution of HA (■) and **HA-FA-HEG-SA** (●) as a function of shear rate (range 1–100 s^−1^).

Self-assembling properties of the polymers were also investigated by evaluating the rheological behavior of **HA-FA-HEG-SA** as a model sample. The viscosity trend of **HA-FA-HEG-SA** vs. shear rate is depicted in Figure 8 and compared with native HA. Both solutions exhibited a shear thinning behavior, since their viscosities decreased with the shear rate increasing. Specifically, **HA-FA-HEG-SA** viscosity decreased from 38 ± 3 mPa∙s to 0.13 ± 0.01 mPa∙s in the shear rate range of 1–100 s^−1^, whereas native HA viscosity decreased from 14 ± 2 mPa∙s to 1.1 ± 0.1 mPa∙s in the same range. The obtained results confirmed the capability of functionalized HA to self-assemble into aggregate structures, whose formation was reflected in the doubled viscosity of the solution in comparison with bare HA.

### 3.4. Olanzapine Loading and Release

Due to the potential ability of the nanosystems to load pharmacologically active molecules, olanzapine [32] was selected as a model drug.

OZ is an antipsychotic agent contained in different pharmaceutical formulations as a free base or hydrochloride salt. It is used for the treatment of central nervous system disorders and is available as coated tablets for oral administration. Recently, the injectable form for intramuscular use of 10 mg was introduced for the emergency treatment of psychomotor agitation in manic crises and schizophrenic patients. OZ shows highly permeability to biological membranes; however, on the other hand, it has very low solubility in water, which limits its bioavailability [25].

In order to increase the bioavailability and reduce its cytotoxic effects, olanzapine was physically adsorbed into both polymeric systems reported above. After incorporation, the systems were purified by centrifugation and filtration in order to remove non-incorporated drug.

Subsequent analysis by DLS showed some modifications of the physical parameters upon drug incorporation, with a consequent lowering of size and the zeta potential with respect to the empty nanosystems (Table 2). Definitively, the calculated drug loading values demonstrated that the NPs are capable of incorporating a good quantity of OZ. Subsequently, release studies were carried out to evaluate the ability of systems to release the incorporated drug.

**Figure 9:**
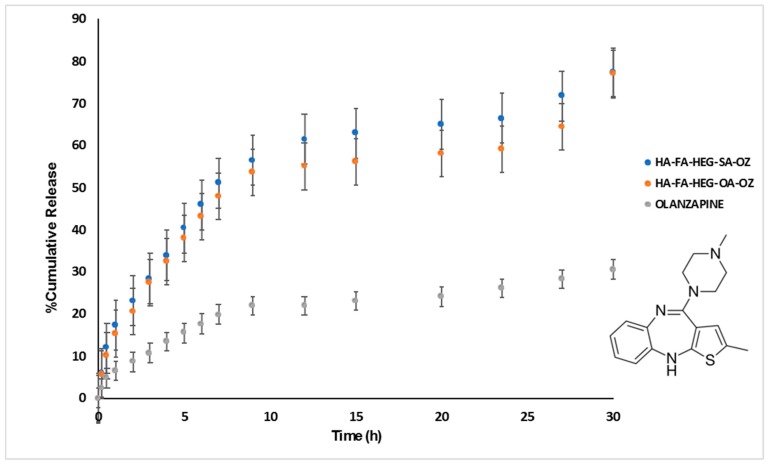
In vitro OZ release profiles in PBS solution pH 7.4 at 37 °C. The amount of released OZ is reported as the percentage ratio between the weight of the drug released from the nano-aggregates and the drug loading.

The release profiles of OZ-loaded NP (Figure 9) suggest that **HA-FA-HEG-OA** and **HA-FA-HEG-SA** aggregates are able to release the loaded active molecules as olanzapine in a controlled way. The assumption on which the release studies were based was a sufficiently dilute solution, able to mimic the donor compartment, where drug solubility does not limit the drug release. However, this situation is difficult to meet with poorly water-soluble drug formulations, as a very large volume of release medium is required with respect to the formulation, making it a challenge to measure the drug concentration accurately [33]. Thus, the low solubility of olanzapine in water required experimental techniques capable of increasing its solubility (in this case, the role of nanoparticles was to increase the solubilization of the drug). In order to alleviate this difficulty, the sampled solution compartment was enriched with a surfactant (i.e., Tween-80 at 3%) with the purpose of increasing the drug solubility. The results shown in the graph of Figure 9 demonstrate the ability of the polymeric systems to improve the release profile of the loaded drug in a defined time as compared to the release profile of the drug alone. In particular, the release of OZ loaded into nanosystems was constant in the first 10 h with a release of about 50% of the encapsulated drug; after 10 h, the release was slower and almost complete (ca. 80%) after 30 h of incubation. By contrast, the release profile of the free drug showed only a 30% release within 30 h.

### 3.5. In Vitro Biological Evaluations

**Figure 10:**
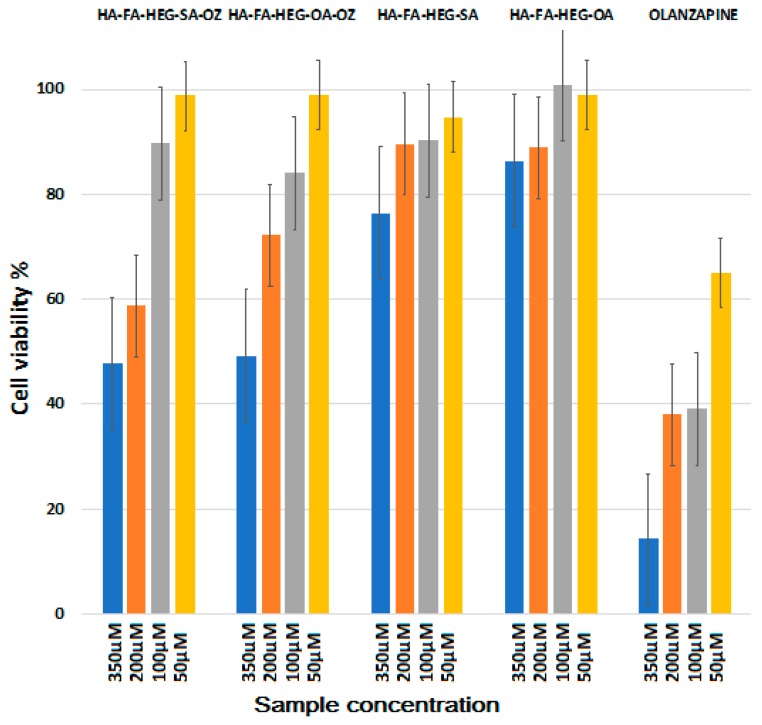
Cell viability evaluated by MTS on 16-HBE cells, after 24 h of incubation with **HA-FA-HEG-OA**, **HA-FA-HEG-SA**, **HA-FA-HEG-OA**-OZ, **HA-FA-HEG-SA**-OZ, and olanzapine alone.

In order to evaluate the possibility of using **HA-FA-HEG-OA** and **HA-FA-HEG-SA** NP as drug release systems in vivo, preliminary biological tests were carried out using the MTS assay [34]. The cytocompatibility of the nanosystems was evaluated using a normal human cell line, namely, bronchial epithelial cells (16HBE) [35]. 16HBE was chosen because it is one of the most commonly used model cell lines for evaluating the cytotoxicity of drugs and toxicants, due to its high sensibility to environmental toxicants [36,37,38]. The studies were carried out in triplicate by incubating the cells for 24 h with empty and OZ-loaded nanosystems at increasing drug concentrations. The results in terms of cell viability (%) are reported in Figure 10. The experiments confirmed that no cytotoxic effects were generated by the empty polymer aggregates showing, in fact, an average cell viability higher than 80%, even at the higher tested concentration. Moreover, we can observe a high cytocompatibility of both OZ-loaded systems at 50 and 100 μM of loaded drug, compared with that of the pure drug. Although the cytocompatibility of the free drug is very low and consequently reduces that of the OZ-loaded systems at 200 and 350 μM, the cell toxicity produced by the latter was attenuated, confirming the possibility of using these nanosystems for further evaluation in vivo at concentrations below 200 μM of loaded drug.

## 4. Conclusions

**Figure 11:**
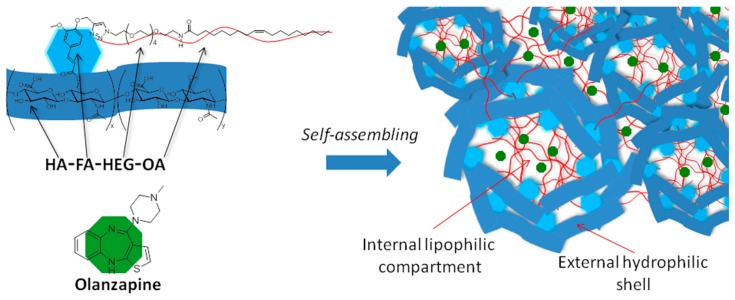
Representation of hyaluronan graft copolymer **HA-FA-HEG-OA** bearing fatty acid residues as self-assembling nanoparticles for olanzapine delivery.

Over the years, pharmaceutical research always showed a great interest in the use of polymeric materials as drug carriers, capable of improving biocompatibility and bioavailability. Among these carriers, polymeric nanoparticles have great potential. In this study, hyaluronan copolymers grafted with propargylated ferulate fluorophores linked to fatty-acid residues by means of hexa(ethylene glycol) spacers, **HA-FA-HEG-OA** and **HA-FA-HEG-SA**, were properly synthesized, characterized, and tested as biocompatible polymeric materials, potentially useful as self-assembled colloidal nanosystems for drug delivery. The obtained nanosystems were characterized from the physico-chemical point of view in terms of average size, zeta potential, and aggregation capacity in an aqueous environment, showing an average size around 180–360 nm, a narrow dimensional distribution, and negative surface charge. The results demonstrated the possibility of using polymeric systems for the production of nano-aggregates capable of delivering hydrophobic drugs such as OZ (Figure 11). Finally, the results of the cytotoxicity test confirmed the absence of cytotoxic effects on a normal human cell line, i.e., bronchial epithelial cells, at concentrations below 200 μM of loaded drug.

The combination of all these positive data offers the advantages of a system that can potentially provide a biocompatible vector for prolonged release of the drug in situ, and it sets the basis for the exploration of other potential applications in the field of drug release control.

## Figures and Tables

**Table 1:** Size distribution and zeta (Z) potential values of empty systems.

Samples	Size (nm)	PDI	Z-potential (mV)
**HA-FA-HEG-OA**	291	0.424	−47
**HA-FA-HEG-SA**	364	0.442	−35

**Table 2:** Size distribution, Z-potential values, and drug loading of drug-loaded systems.

Samples	Size (nm)	PDI	Z-Potential (mV)	Drug Loading (%)
**HA-FA-HEG-OA-OZ**	234	0.352	−31.1	14.0
**HA-FA-HEG-SA-OZ**	178	0.260	−24.7	14.2
